# Fibroblast Growth Factor (FGF-2) and Its Receptors FGFR-2 and FGFR-3 May Be Putative Biomarkers of Malignant Transformation of Potentially Malignant Oral Lesions into Oral Squamous Cell Carcinoma

**DOI:** 10.1371/journal.pone.0138801

**Published:** 2015-10-14

**Authors:** Seema Nayak, Madhu Mati Goel, Annu Makker, Vikram Bhatia, Saumya Chandra, Sandeep Kumar, S. P. Agarwal

**Affiliations:** 1 Department of Pathology, King George’s Medical University, Lucknow, U.P. - 226003, India; 2 All India Institute of Medical Sciences Bhopal, M.P. – 462026, India; 3 Department of Otorhinolaryngology, King George’s Medical University Lucknow, U.P. – 226003, India; China Medical University, TAIWAN

## Abstract

There are several factors like angiogenesis, lymphangiogenesis, genetic alterations, mutational factors that are involved in malignant transformation of potentially malignant oral lesions (PMOLs) to oral squamous cell carcinoma (OSCC). Fibroblast growth factor-2 (FGF-2) is one of the prototypes of the large family of growth factors that bind heparin. FGF-2 induces angiogenesis and its receptors may play a role in synthesis of collagen. FGFs are involved in transmission of signals between the epithelium and connective tissue, and influence growth and differentiation of a wide variety of tissue including epithelia. The present study was undertaken to analyze expression of FGF-2 and its receptors FGFR-2 and FGFR-3 in 72 PMOLs, 108 OSCC and 52 healthy controls, and their role in risk assessment for malignant transformation of Leukoplakia (LKP) and Oral submucous fibrosis (OSMF) to OSCC. Immunohistochemistry was performed using antibodies against FGF-2, FGFR-2 and FGFR-3. IHC results were validated by Real Time PCR. Expression of FGF-2, FGFR-2 and FGFR-3 was upregulated from PMOLs to OSCC. While 90% (9/10) of PMOLs which showed malignant transformation (transformed) expressed FGF-2, only 24.19% cases (15/62) of PMOLs which were not transformed (untransformed) to OSCC expressed FGF-2. Similarly, FGFR-2 expression was seen in 16/62 (25.81%) of untransformed PMOLs and 8/10 (80%) cases of transformed PMOLs. FGFR-3 expression was observed in 23/62 (37.10%) cases of untransformed PMOLs and 6/10 (60%) cases of transformed PMOLs. A significant association of FGF-2 and FGFR-2 expression with malignant transformation from PMOLs to OSCC was observed both at phenotypic and molecular level. The results suggest that FGF-2 and FGFR-2 may be useful as biomarkers of malignant transformation in patients with OSMF and LKP.

## Introduction

Oral squamous cell carcinoma (OSCC) is a multifactorial and complex disease. Several factors including angiogenesis, lymphangiogenesis, alterations in expression or structure of tumor suppressor genes and oncogenes and their proteins, etc are involved in malignant transformation of potentially malignant oral lesions (PMOLs) to OSCC [1, 2].

The malignant transformation rates of PMOLs show a great variation; for example, 10–20% of hyperkeratosis or epithelial hyperplasia or epithelial dysplasia may transform to cancer and the estimated annual rate is 1.4–7% [3, 4]. Lumerman in 1995 reported that 6.6%–36% of epithelial dysplasias may transform to invasive SCC [5]. The malignant transformation rate of leukoplakia (LKP) has been reported to range from 0.13 to 17.5% [6,7], 1.1% in Oral lichen planus [8] and 2.3–7.6% in oral submucous fibrosis (OSMF), during 10–17 years of follow-up [9,10]. In a study from Taiwan [11], the malignant transformation rate in an average follow-up period of 42.6 months ranged from 1.9 to 5.4% for various types of PMOLs.

At present, histologic assessment of epithelial dysplasia is the gold standard for determining the malignant transformation risk of PMOLs. However, the problem of inter and intra observer variation is always there in the histopathologic assessment of presence and severity of epithelial dysplasia [12, 13]. In spite of tremendous progress in the field of molecular biology, there is as yet no single marker or set of markers that reliably predict malignant transformation of LKP in individual patients [14]. Therefore, objective biomarkers which do not require the ability to recognize morphologic changes are needed to evaluate the risk of malignant transformation of PMOLs.

FGF-2 is one of the prototypes of a large family of growth factors which is expressed in several tissues and has a wide scope of biologic activities [15]. It binds to low affinity heparin sulfate proteoglycans that are involved in the interaction with high affinity receptors that in turn mediate the cellular response to FGF-2 [16, 17]. The FGF receptor family consists of four members (FGFR-1(flg), FGFR-2 (bek), FGFR-3 and FGFR-4) that have 55–72% amino acid homology [18].

FGF-2 induces angiogenesis [19, 20, 21] and its receptors may play a role in synthesis of collagen. It is involved in the transmission of signals between the epithelium and connective tissue, and influences growth and differentiation of a wide variety of tissue including epithelia [22].

Studies have reported FGF-2 overexpression in high grade malignant tumours and malignant transformation of normal cells transfected with FGF-2 gene [23, 24]. FGF-2 is involved in the invasion of cancer cells and the proliferation of fibroblasts around cancer cells in an autocrine or paracrine fashion [25]. The study of expression of this factor in head and neck carcinomas (HNC) has yielded controversial results [26, 27, 28, 29]. It has been suggested that deployment of FGFR-specific tyrosine kinase inhibitors as single agent or in combination with EGFR inhibitors may be one of the effective therapeutic strategies in Head and Neck squamous cell carcinoma [30].

The aim of present study was to analyze the expression of FGF-2 and its receptors FGFR-2 and FGFR-3 in PMOLs and OSCC and their role in risk assessment for malignant transformation of LKP and OSMF to OSCC.

## Materials and Methods

### Subjects and sample collection

Tissue biopsies were obtained from cases of 72 PMOLs which included 43 cases of LKP and 29 cases of OSMF, 108 OSCC and 52 healthy controls from the Departments of Oral and Maxillofacial Surgery and Otorhinolaryngology, King George’s Medical University Lucknow after obtaining the Institutional Ethical approval and informed written consent from patients during the year 2007 to 2012. Healthy oral tissues were obtained from patients undergoing cosmetic surgery, who otherwise did not have any infective or inflammatory oral lesion. A small part of the tissue was snap frozen for molecular work and was stored at -80°C. The inclusion criteria followed for PMOLs patients was as described by Ho et al. (2009) [31]. Relevant clinical and demographic details of each patient were recorded on a structured proforma.

### Histopathological examination

All tissues were fixed in 10% neutral buffered formalin and processed for histopathological examination as per standard procedure. 5μm thick sections were cut and stained with haematoxylin and eosin (H&E). Sections were reviewed by two independent pathologists and histological diagnosis was made as per WHO criteria and following the classification by Warnakulasuriya et al 2007 [32] where the clinically diagnosed cases of leukoplakia on histopathology of tissue biopsies, are divided into leukoplakia with dysplasia and leukoplakia without dysplasia. Among 29 cases of the OSMF group in the present study, 11 were without dysplasia, 4 showed mild dysplasia, 5 with moderate dysplasia and 9 cases showed severe dysplasia. Among 43 cases in the Leukoplakia group, 10 cases showed no dysplasia, 3 had mild dysplasia, 10 with moderate dysplasia and 20 cases showed severe dysplasia.

The patients were followed up every 2 months in the first year, every 3 months in the second year, and every 4 to 6 months thereafter. The patients were followed to a maximum of 5 years. Between the follow ups some patients with leukoplakic lesions and OSMF without dysplasia, underwent treatment at an early stage and were discontinued from the follow-up.

### Immunohistochemistry

Sections were deparaffinized in xylene followed by hydration in descending ethanol grades. Endogenous peroxidase was blocked in 3% H_2_O_2_ in methanol for 30 min. Antigen retrieval was performed by heating specimens for 15 min at 95°C in citrate buffer (pH 6.0) using an EZ antigen retriever system (BioGenex, USA). Sections were then incubated with power block (BioGenex, USA) for 10 min to reduce nonspecific antibody binding. The sections were incubated overnight at 4°C with primary antibodies. Mouse monoclonal antibodies against human FGF-2, FGFR-2 and FGFR-3 (Santa Cruz Biotechnology Inc., Santa Cruz, CA) were used. Primary antibodies were detected using super sensitive polymer-HRP IHC detection system (BioGenex, USA). After thorough washing with Tris buffered saline (TBS; pH 7.4) sections were treated with super enhancer for 20 min at room temperature followed by incubation with poly-HRP reagent for 30 min at room temperature. After three washes with TBS, DAB substrate (3, 3’-diaminobenzidine tetra hydrochloride) was applied to the sections for 5–10 min in the dark. Sections were counterstained with hematoxylin, dehydrated with ascending ethanol grades and xylene and mounted permanently with DPX. Negative control sections were processed by omitting primary antibody and breast carcinoma tissue was used as positive control.

### Evaluation of staining

The level of expression was assessed by semiquantitative scoring which included the overall percentage area of the lesion stained positive (0–100%), and the staining intensity (0–3). In all the cases, the expression in epithelium, endothelial cells and stroma was analyzed. Grading for percentage area positivity was done as follows: <10% = 0, 10–25% = 1, 25–50% = 2, 50–75% = 3, >75 = 4. To evaluate the intensity, grading was done as; 0 = none, 1 = mild, 2 = moderate, 3 = strong staining. The percentage score (0–4) was multiplied by the intensity score (0–3) and a final score was assigned, 0–4 as negative staining, 5–12 as positive staining [33]. At least five best fields were taken for interpreting results of percentage area.

### Quantitative real-time PCR (qPCR) for *FGF-2*, *FGFR-2* and *FGFR-3* genes

#### Total RNA extraction

RNA was extracted from frozen tissue samples with Trizol reagent (Invitrogen, Carlsbad, CA). RNA purification was done by DNase1 treatment (Invitrogen, Amplification grade). In brief, 1μg of total RNA sample was treated with 10X DNase I reaction buffer and DNase I (1U/10μl) and incubated for 15 min at room temperature followed by inactivation of DNase I with 25mM EDTA at 65°C for 10 min. RNA was quantified by Qubit 2.0 fluorometer (Molecular Probes, Invitrogen, USA).

#### cDNA synthesis

250 ng of the total RNA was subjected to reverse transcription using random hexamer primers with Gene AMP RNA PCR kit (Applied Biosystems, Foster city, CA) for cDNA synthesis, as per manufacturer’s instructions. Briefly, the 20μl reaction was performed in 3 steps. Step 1 at 25°C for 10 min, step 2 at 37°C for 2 hrs and finally step 3 at 85°C for 5min. cDNA was stored at -20°C for real time PCR.

#### Quanitative real time PCR (qPCR)

qPCR was performed using StepOne Real-time PCR system (Applied Biosystems, USA) in the presence of SYBR Green fluorescent dye according to the manufacturer’s instructions. Briefly, 20μl of the reaction mixture consisting of reverse transcribed cDNA, 2X SYBR Green master mix containing dNTPs, ROX dye and 10μM of forward and reverse primers was dispensed into a fast optical 48-well real time PCR reaction plate (Applied Biosystems, USA). The PCR primers for *FGF-2* [34], *FGFR-2* [34], and *FGFR-3* [30] genes were selected from a published article and synthesized by MWG, India. Primer sequences were rechecked using Primer Express software 3.0 (Applied Biosystems, USA) and checked for homology by Blast sequence analysis (National Centre for Biotechnology Information). Following primers sequences were used: β-actin (endogenous control): forward 5’-GAGACCTTCAACACCCCAGCC-3’; reverse 5’-AGACGCAGGATGGCATGGG-3’, FGF-2: forward5’-ATGGCAGCCGGGAGCATCACCCACG-3’; reverse: 5’TCAGCTCTTCGCAGACATTGGAAG-3’, FGFR-2: forward 5’-TCCACATGGAGATATGGAACAGGA-3’; reverse 5’-GGAGCTATTTATCCCCGAGTG-3’ FGFR-3 forward 5’- CCATCGGGCATTGACAAGGAC-3’, reverse 5’- GCATCGTCTTTCAGCATCTTCAC-3’. Thermal cycle conditions consisted of on initial denaturation incubation at 95°C for 10min followed by 40 cycle of 15sec incubation at 95°C and 60sec incubation at 60°C followed by the thermal dissociation (melt curve) protocol for fluorescence detection. Gene expression level was determined using the 2^-ΔΔCt^ method using beta-actin as an endogenous control. A negative control without a template was run in parallel to assess the overall specificity of the reaction. All reactions were run in replicates. Data are presented as “relative gene expression”.

### Statistical analysis

Statistical analysis was performed using version 17.0 SPSS software for windows (SPSS, INC, Chicago, IL). For assessing proportional data, chi-square test was carried out. Correlation between FGF-2, FGFR-2 & FGFR-3 was determined by Spearman correlation coefficient. Logistic regression was applied to evaluate hazard ratios for the malignant transformed PMOLs. Odds ratios (OR) with 95% confidence interval (95% CI) was calculated and p-values were reported. For all the tests, a p-value < 0.05 was considered statistically significant.

## Results

### Clinicopathological features

The study population comprised of 172 (74.14%) males and 60 (25.86%) females with a median age of 42 years (range 8 to 85 years). In PMOL group dysplasia was present in 51 out of 72 cases. In OSCC group, histologically 8 cases were poorly differentiated, 25 cases were moderately differentiated and 75 cases were well differentiated. 62 tumors were clinical stage I or II, and 46 tumors were clinical stage III or IV. Lymph node involvement was found in 41 cases of OSCC.

### Immunohistochemical expression of FGF-2 and its receptors (FGFR-2 and FGFR-3) in PMOLs and OSCC

**Fig 1:**
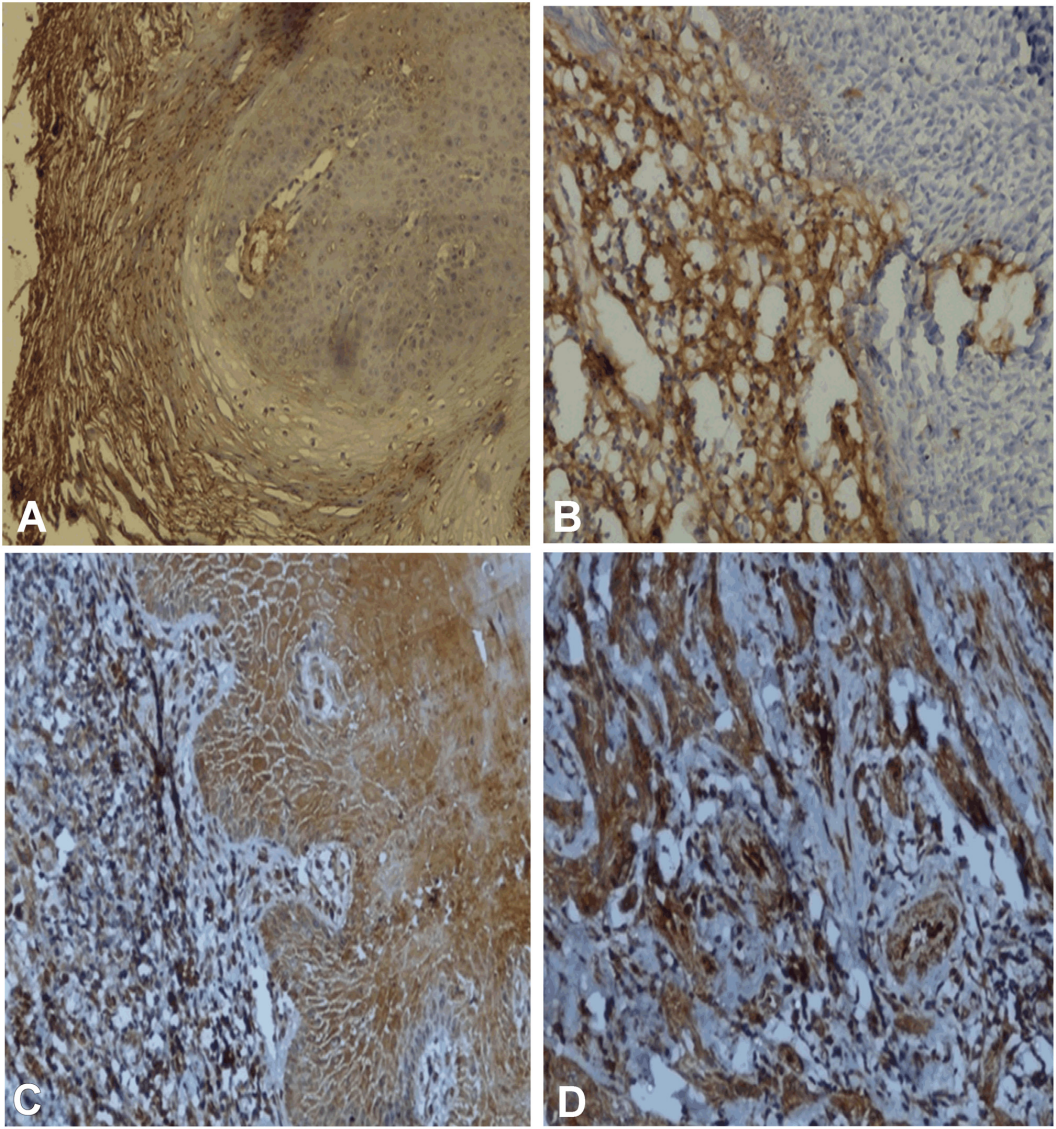
Immunohistochemical staining of FGF-2. (A-D). A, FGF-2 positivity in transformed case of Leukoplakia with dysplasia X 100. B, FGF-2 positivity in transformed cases of OSMF in fibrosis area X 100. C, FGF-2 positivity in epithelium of OSCC X 400. D, FGF-2 positivity in tumor cells OSCC X 400.

**Fig 2:**
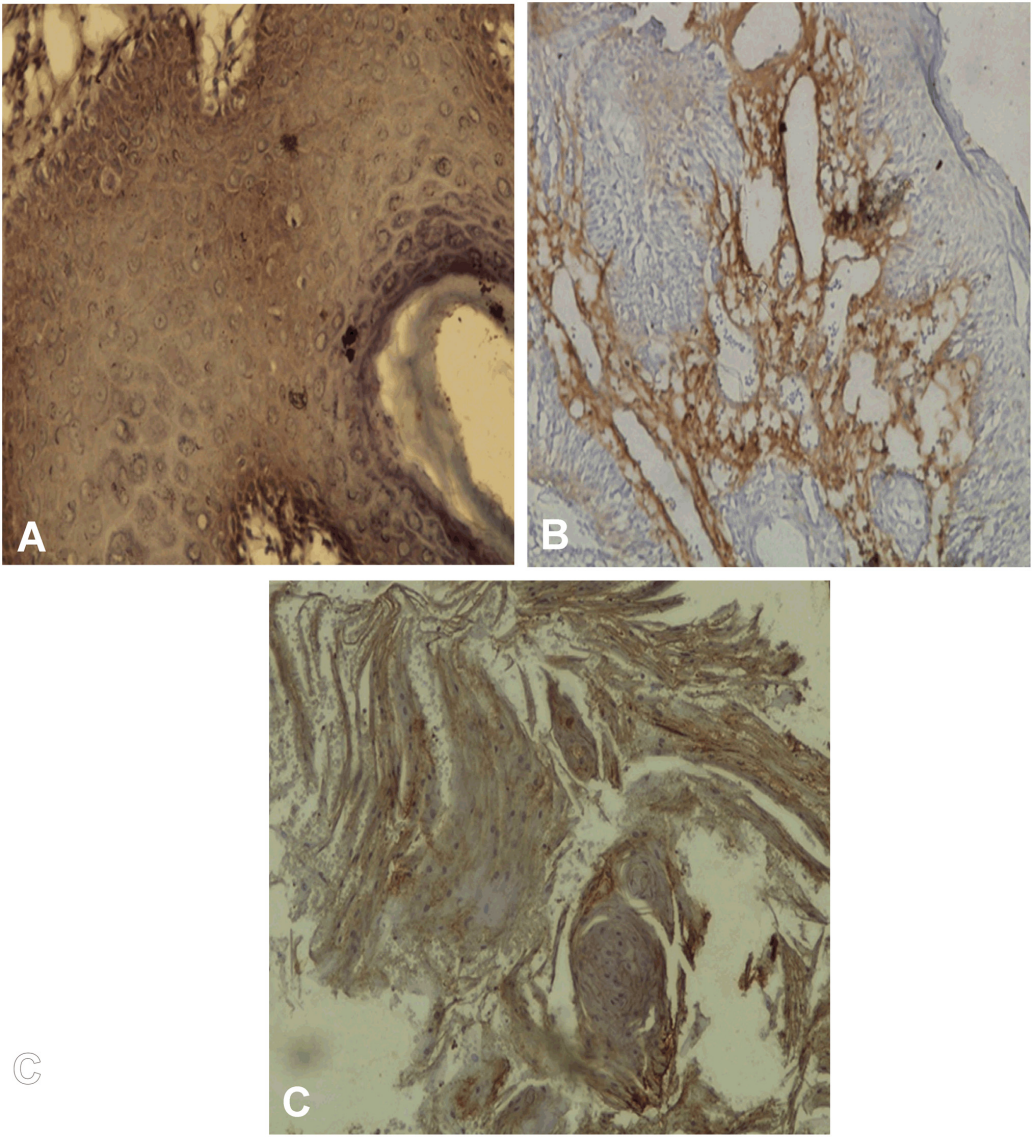
Immunohistochemical staining of FGFR-2 (A-C). A, FGFR-2 positivity in transformed cases of Leukoplakia with dysplasia X 200. B, FGFR-2 positivity in transformed case of OSMF in fibrosis area X 100. C, FGFR-2 positivity in OSCC X 100.

**Fig 3:**
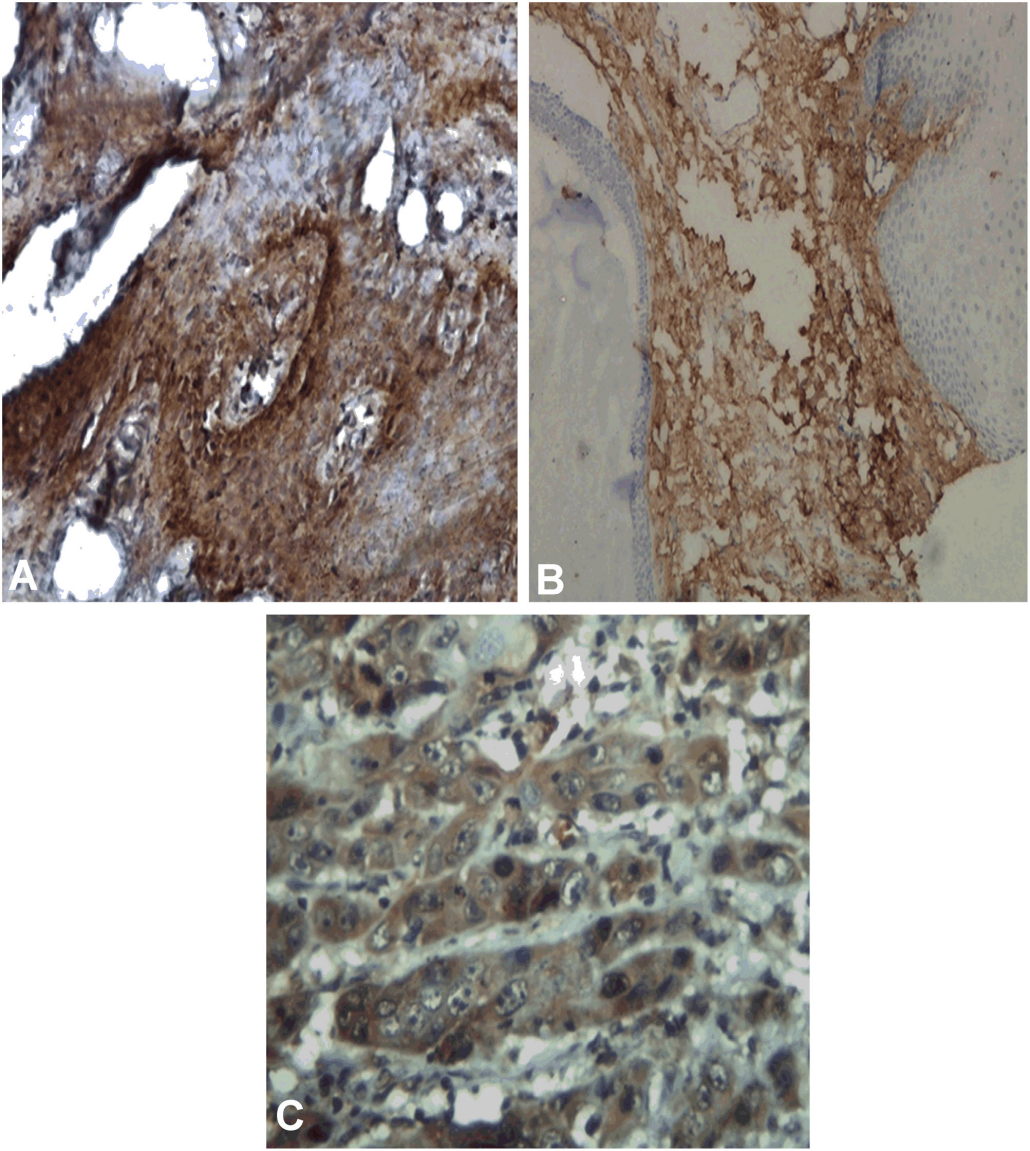
Immunohistochemical staining of FGFR-3 (A-C). A, FGFR-3 positivity in epithelium of Leukoplakia with dysplasia in transformed case X 200. B, FGFR-3 positivity in transformed case of subepithelial fibrosis area of OSMF X 100. C, FGFR-3 positivity in tumor cells of OSCC X 400.

Immunhistochemical expression of FGF-2, FGFR-2 and FGFR-3 is shown in Table 1. Expression of FGF-2 was cytoplasmic in basal, parabasal layers in tissues with lining epithelium, tumor cells and also in stroma as shown in “Fig 1A, 1B, 1C and 1D”. FGF-2 positivity was observed in 49.08% (53/108) OSCC cases, 31.95% (23/72) PMOLs and in 26.92% (14/ 52) control cases. Expression of FGFR-2 was found to be cytoplasmic in full thickness of epithelium, tumor cells and stromal cells as shown in “Fig 2A, 2B and 2C”. FGFR-2 expression was observed in 57.41% (62/108) cases of OSCC, 33.33% (24/72) cases of PMOLs and 15.38% (8/52) cases of controls. Cytoplasmic FGFR-3 expression was observed in upper and midstratum layer of lining epithelium, stromal fibroblast cells, tumor cells and seldom in endothelium of tumor stroma as shown in “Fig 3A, 3B and 3C”. 75% cases (81/108) of OSCC, 40.28% cases (29/72) of PMOLs and 36.54% (19/52) controls stained positive for FGFR-3.

**Table 1:** Immunohistochemical expression of FGF-2, FGFR-2 and FGFR-3 in OSCC and PMOLs.

Groups	FGF2Negative	FGF-2 Positive	P -value	FGFR-2Negative	FGFR-2 Positive	P -value	FGFR-3Negative	FGFR-3 Positive	P -value
	N (%)	N (%)		N (%)	N (%)		N (%)	N (%)	
**Controls**	38(73.0)	14(26.92)		44(84.62)	8(15.38)		33(63.46)	19(36.54)	
**PMOLs**	49(68.0)	23(31.95)	0.445	48(66.67)	24(33.33)	**0.024**	43(59.72)	29(40.28)	0.428
OSMF	20(68.9)	9(31.03)	0.694	22(75.86)	7(24.14)	0.331	18(62.07)	11(37.93)	0.282
LKP	29(67.4)	14(32.56)	0.402	26(60.47)	17(39.53)	**0.008**	25(58.14)	18(41.86)	0.296
**OSCC**	55(50.92)	53(49.08)	**0.008**	46(42.59)	62(57.41)	**0.000**	27(25)	81(75)	**0.002**

### Association of FGF-2, FGFR-2 and FGFR-3 with clinicopathological parameters in PMOLs and OSCC

Association of FGF-2, FGFR-2 and FGFR-3 with clinicopathological parameters in PMOLs and OSCC is shown in Tables 2 and 3. FGF-2 expression was significantly associated with sex (p<0.013) and tumor differentiation (p<0.0001) in OSCC patients. While, FGFR-2 was not associated with any clinicopathological features in oral cancer, FGFR- 3 expression was significantly higher (p<0.043) in stages I-II. No association of FGF-2, FGFR-2 and FGFR-3 was found with clinicopathological parameters in PMOLs.

**Table 2:** Relation of FGF-2, FGFR-2 and FGFR-3 expression with clinico-pathological parameters in PMOLs.

Variables	FGF-2positive	FGF-2 negative	P-value	FGFR-2positive	FGFR-2negative	P-value	FGFR-3positive	FGFR-3negative	P-value
**Age**									
<42	16	35	0.582	15	36	0.271	21	30	0.809
≥42	8	13		9	12		8	13	
**Sex**									
Male	17	37	0.564	20	34	0.248	21	33	0.677
Female	7	11		4	14		8	10	
**Dysplasia**									
Present	19	32	0.271	17	34	1.000	18	33	0.179
Absent	5	16		7	14		11	10	
**Tobacco chewing habit**									
Present	16	32	1.000	16	32	1.000	20	28	0.734
Absent	8	16		8	16		9	15	
**Alcohol**									
Present	8	20	0.494	10	18	0.732	9	19	0.262
Absent	16	28		14	30		20	24	
**Smoking**									
Present	12	24	1.000	10	26	0.317	12	24	0.230
Absent	12	24		14	22		17	19	

**Table 3:** Association of FGF-2, FGFR-2 and FGFR-3 with clinico-pathological features in OSCC.

Variables	FGF-2 positive	FGF-2Negative	P-value	FGFR-2positive	FGFR-2negative	P-value	FGFR-3positive	FGFR-3negative	P-value
**Age**									
<42	14	16	0.756	19	11	0.440	26	4	0.082
≥42	39	39		43	35		55	23	
**Sex**									
Male	49	41	**0.013**	55	35	0.080	69	21	0.371
Female	4	14		7	11		12	6	
**Lymph node**									
Positive	16	25	0.102	20	21	0.156	27	14	0.086
Negative	37	30		42	25		54	13	
**Differentiation**									
WD	32	43	**0.000**	40	35	0.069	52	23	0.077
MD	19	6		19	6		23	2	
WD	2	6		3	5		6	2	
**Tumor stage**									
Stage I-II	31	31	0.823	31	31	0.071	42	20	**0.043**
StageIII-IV	22	24		31	15		39	7	
**Tobacco chewing habit**									
Present	43	41	0.410	50	34	0.405	61	23	0.285
Absent	10	14		12	12		20	4	
**Alcohol**									
Present	28	24	0.339	27	25	0.267	37	15	0.374
Absent	25	31		35	21		44	12	
**Smoking**									
Present	46	44	0.344	50	40	0.384	67	23	0.766
Absent	7	11		12	6		14	4	

### Quantitative real time PCR for *FGF-2*, *FGFR-2* and *FGFR-3*

**Fig 4:**
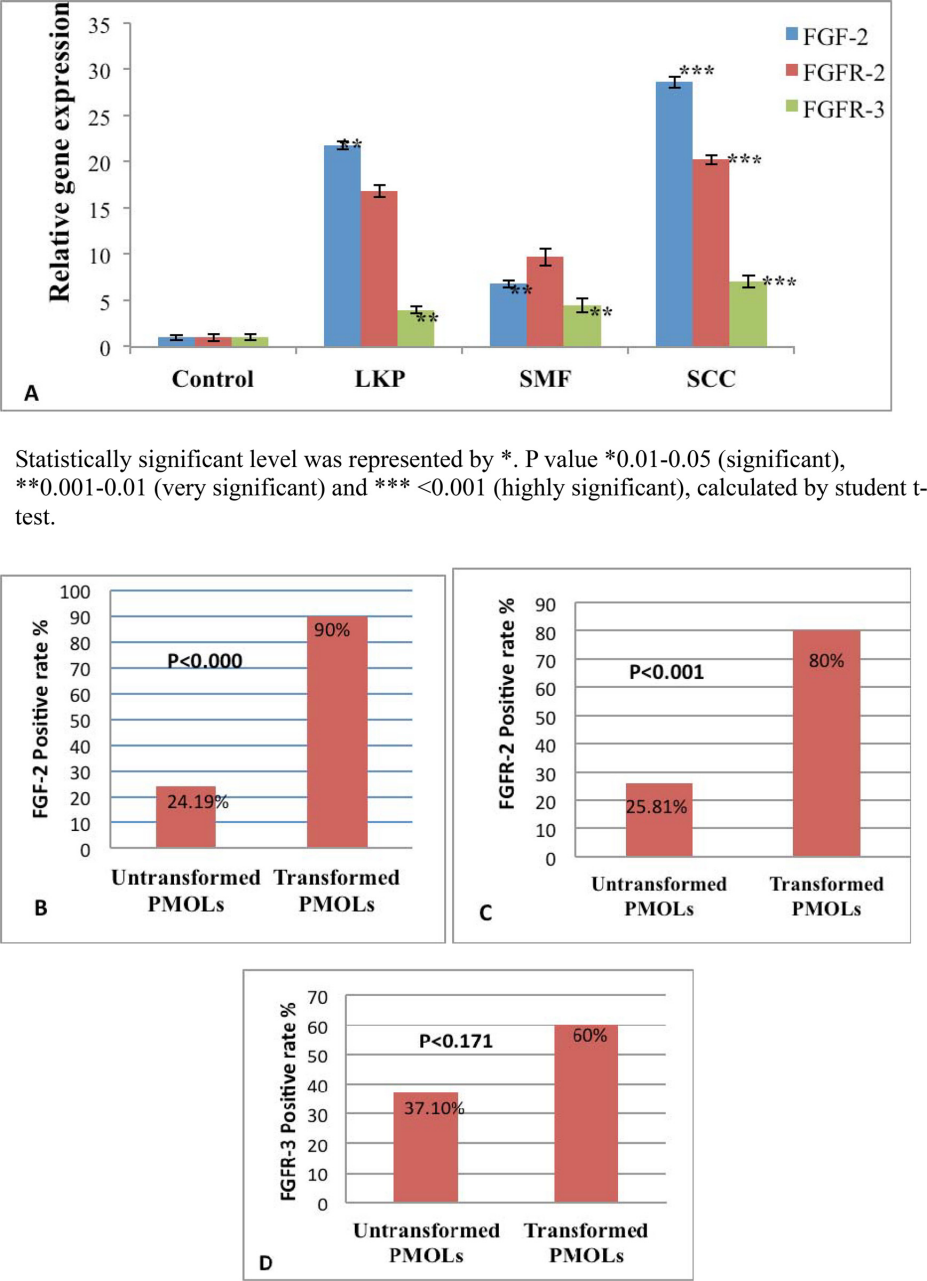
Real time PCR results of FGF-2, FGFR-2 and FGFR-3 and frequency of FGF-2, FGFR-2 and FGFR-3 malignant transformation rate (A-D). Bar diagram showing fold change expression of FGF-2, FGFR-2 and FGFR-3 by Real Time. **A**, PCR in OSMF, LKP, OSCC and healthy controls. B, Frequency of FGF-2 malignant transformation rate in PMOLs. C, Frequency of FGFR-2 malignant transformation rate in PMOLs. D, Frequency of FGFR-3 malignant transformation rate in PMOLs.

Real time PCR was performed to validate the results of immunohistochemistry as shown in Table 4, “Fig 4A”. The same tissue specimens that overexpresed FGF-2 and receptors by IHC were subjected to PCR.

**Table 4:** Gene expression profile of *FGF-2*, *FGFR-2* & *FGFR-3* by Quantitative Real—time PCR.

Gene	Controls(N = 11)	LKP(N = 8)	OSCC(N = 11)	OSMF(N = 29)
***FGF-2***				
Fold Change	1	21.75	28.62	6.79
SE	0.2730769	0.45445	0.6142065	0.3886392
P-value		**0.002**	**0.000**	**0.001**
***FGFR-2***				
Fold Change	1	16.79	20.21	9.72
SE	0.3936878	0.6452975	0.496684	0.9335131
P-value		0.065	**0.000**	0.322
***FGFR-3***				
Fold Change	1	4.45	7.05	3.95
SE	0.333856	0.7594367	0.6345427	0.3587593
P-value		**0.008**	**0.000**	**0.005**

We found that OSCC patients had significantly higher level of FGF-2, FGFR-2 and FGFR-3 expression followed by LKP and OSMF. In OSCC patients relative gene expression of FGF-2 was 28.62 fold higher, FGFR-2 was 20.21 fold higher and FGFR-3 was 7.05 fold higher as compared to healthy controls.

### Correlation between FGF-2, FGFR-2 and FGFR-3 in PMOLs and OSCC

In PMOLs, FGF-2 was significantly correlated with FGFR-2 (rs = 0.313, p<0.008). FGFR-2 showed significant correlation with FGFR-3 (rs = 0.260, p<0.027). In OSCC, FGF-2 was significantly correlated with FGFR-2 (rs = 0.321, p<0.001) and FGFR-3 (rs = 0.353, p<0.000). As in PMOLs, FGFR-2 showed significant correlation with FGFR-3 (rs = 0.368, p<0.000).

### Malignant transformation (from PMOLs to OSCC) and association with FGF-2, FGFR-2 and FGFR-2

Malignant transformation of PMOLs to OSCC was observed in 13.89% (10/72) cases (3 OSMF and 7 LKP cases) as shown in Table 5. Some cases of both OSMF (n = 3) and LKP (n = 4) groups, with either no dysplasia or mild dysplasia regressed after the course of treatment.

**Table 5:** Patients characteristic of malignant transformed and untransformed cases in PMOLS.

Patients Characteristics	Untransformed PMOLs (N = 62)	Malignant transformed PMOLs (N = 10)	P-value
**Age (Years)**			
Mean ± SD	37.81± 11.98	43.60± 18.65	0.383
Range	20–80	22–75	
**Sex N (%)**			
Female	17 (27.42%)	1 (10%)	0.238
Male	45 (72.58%)	9 (90%)	
**Dysplasia**			
Present	41 (66.13%)	10 (100%)	**0.029**
***Mild***	7	0	
***Moderate***	15	0	
***Severe***	19	10	
Absent	21 (33.87%)	0 (0%)	
**Tobacco chewing habit**			
Present	40 (64.52%)	8 (80%)	0.335
Absent	22 (35.48%)	2 (20%)	
**Alcohol**			
Present	24 (38.71%)	4 (40%)	0.938
Absent	38 (61.29%)	6 (60%)	
**Smoke**			
Present	29 (46.77%)	7 (70%)	0.173
Absent	33 (53.23%)	3 (30%)	
**Follow—up**			
Mean	26.21	29.41	0.749
Range(months)	3–55	10–60	
**FGF-2 expression**			
Negative	47 (75.81%)	1 (10%)	**0.000**
Positive	15 (24.19%)	9 (90%)	
**FGFR-2 expression**			
Negative	46 (74.19%)	2 (20%)	**0.001**
Positive	16 (25.81%)	8 (80%)	
**FGFR-3 expression**			
Negative	39 (62.90%)	4 (40%)	0.171
Positive	23 (37.10%)	6 (60%)	

FGF-2 expression was seen in 15/62 cases (24.19%) of untransformed PMOLs and 9/10 cases (90%) of transformed PMOLs as shown in “Fig 4B”. FGFR-2 expression was observed in 16/62 (25.81%) of untranformed PMOLs and 8/10 (80%) transformed PMOLs as shown in “Fig 4C”. FGFR-3 expression was observed in 23/62 (37.10%) cases of untransformed PMOLs and 6/10 cases (60%) in transformed PMOLs as shown in “Fig 4D”. The characteristics of individual malignant transformed cases are described in Table 6. FGF-2 and FGFR-2 expression was found to be significantly associated with malignant transformation of PMOLs to OSCC both at phenotypic (FGF-2 p<0.000, FGFR-2 p<0.001), and molecular level (FGF-2 p<0.019, FGFR-2 p<0.025). No association of FGFR-3 was found with malignant transformation.

**Table 6:** Characteristics of the malignant transformed cases.

CaseNo.	Age	Sex	Diagnosis	Site of lesion	Tobacco chewing habit	Alcohol	Smoking	dysplasia	FGF-2	FGFR-2	FGFR-3	Follow- up in months
1	25	M	SMF	BM	P	A	A	P	A	A	P	40
2	22	M	SMF	BM	P	A	P	P	P	P	P	60
3	60	M	SMF	BM	P	A	A	P	P	P	A	18
4	34	M	LKP	T	A	P	P	P	P	P	P	34
5	55	F	LKP	BM	P	A	P	P	P	P	P	39
6	65	M	LKP	BM	P	A	A	P	P	P	A	29
7	30	M	LKP	G	P	P	P	P	P	P	A	22
8	40	M	LKP	BM	P	P	P	P	P	A	A	23
9	75	M	LKP	BM	P	P	P	P	P	P	P	10
10	30	M	LKP	BM	A	A	P	P	p	p	P	18

M, Male; F, Female; BM, Buccal mucosa; T, Tongue; G, Gingival buccal sulcus. P, Present; A, Absent.

### Logistic regression analysis of malignant transformation risk in PMOLs

To evaluate the risk of malignant transformation of PMOLs we analyzed clinicopathological parameters and FGF-2 and FGFR-2 expression by logistic regression. In the univariate regression analysis, FGF-2 and FGFR-2 expression was associated with 28.2-fold (95% CI, (3.29–241.72); P <0.002) and 11.5-fold (95% CI, 2.20–59.91; P <0.004) increased risk of malignant transformation, respectively as shown in Table 7. To further assess the influence of each factor, we did multivariate regression analysis. Both these factors retained statistical significance. The OR for transformation was 19.91 for FGF-2 (95% CI, 12.20–180.32; P <0.008) and 7.22 for FGFR-2 (95% CI, 1.20–43.31; P <0.031). Interestingly, when co-expression of FGF-2 and FGFR-2 was considered as cofactor, the risk of malignant transformation was considerably higher in PMOLs showing co-expression as compared to PMOLS without co-expression of FGF-2and FGFR-2 (OR, 4.19; 95% CI, 1.81–9.71; P < 0.001).

**Table 7:** Logistic regression analysis of variables in PMOLs malignant transformation.

Variables	OR (95% CI)	P-value
**Univariate analysis**		
Age	1.03 (.98–1.08)	0.201
Sex	0.29 (.03–2.50)	0.262
Tobacco	2.20 (.42–11.27)	0.344
Alcohol	1.05 (.27–4.13)	0.938
Smoking	2.65 (.62–11.22)	0.184
FGF-2 expression	28.20 (3.29–241.72)	**0.002**
FGFR-2 expression	11.5 (2.20–59.91)	**0.004**
FGFR-3 expression	2.54 (.64–9.97)	0.180
**Multivariate analysis**		
FGF-2 expression	19.91 (2.20–180.32)	**0.008**
FGFR-2 expression	7.22 (1.20–43.31)	**0.031**
**Coexpression of FGF-2/FGFR-2**	4.19 (1.81–9.71)	**0.001**

OR, Odds ratio; 95%CI, 95% confidence interval.

## Discussion

The transformation of oral precancerous lesions to cancer is a known fact. However it is difficult to predict which precancerous lesions will progress into malignancy. The present study evaluated the expression of FGF-2 and its receptors FGFR-2 and FGFR-3 in PMOLs and OSCC and their role in assessment for malignant transformation of PMOLs to OSCC.

FGF-2 is involved in induction of angiogenesis in several cancers; phaeochromocytoma, renal cell carcinoma, astrocytoma, bladder carcinoma, hepatocellular carcinoma, and prostate cancer [35] and also in the signals between the epithelium and connective tissue, influencing growth and differentiation. We observed expression of FGF-2 and its receptors FGFR-2 and FGFR-3 in neoplastic progression from normal through stages of epithelial dysplasia to oral squamous cell carcinoma. FGF-2 immunohistochemical expression was observed in 49.08% (53/108) cases of OSCC, 31.95% (23/72) cases of PMOLs and 26.92% (14/52) of controls. The expression of FGF-2 was cytoplasmic in basal and parabasal layers with lining squamous epithelium, tumor cells and also in the tumor stroma. Our findings are similar to that of Raimondi et al. (2006) [36] who have reported FGF-2 immunostaining only in cytoplasm of the basal layers of the epithelium in hamster cheek pouch model of oral cancer. Higher expression of FGF-2 in breast cancer stroma as compared to normal breast stroma has been reported [37].

FGF-2, besides being expressed in cancer and precancer, was also found in 26.92% cases of healthy controls in the present study. Wakulich et al (2002) [28] noticed weak to intense staining of FGF-2 in basal and parabasal layers in normal epithelium of 78.57% (11/ 14) cases. They found no statistically significant difference in staining intensity between normal and carcinomatous tissue. Further the presence of FGF-2 expression in basal and parabasal layers suggested that this growth factor was involved in proliferation but not in differentiation of normal oral keratinocytes.

FGF-2 expression was significantly high (60.38%) in well differentiated tumors in the present study. Janot et al. (1995) [26] and Wakulich et al. (2002) [28] have also reported increased FGF-2 staining in well differentiated tumors suggesting that it may be involved in mitosis seen in dysplasia, carcinoma in situ and OSCC.

In the present study, FGFR-2 expression was observed in 57.41% (62/108) cases of OSCC, 33.33% (24/72) cases of PMOLs and 15.38% (8/52) cases of controls. Wakulich et al. (2002) [28] have also reported generalized increase in staining intensity at all levels in dysplasias and carcinomatous tissue. While we observed FGFR-2 expression in normal epithelium in only 8 control cases, Dellacono et al. (1997) [27] found positive FGFR-2 staining in normal epithelium as well as in patches in OSCC. Forootan et al. (2000) [38] have reported increased FGFR-2 expression in superficial layers of normal epithelium and in areas of keratinization in squamous cell carcinomas. Recently, overexpression of FGFR-2 in breast cancer cell lines was reported to lead to constitutive FGFR-2 activation. Inhibition of FGFR-2 signaling in these cells induced apoptosis [38]. Thus, constitutive FGFR-2 signaling due to FGFR-2 overexpression can lead to protection from apoptosis which is one of the hallmarks of cancer [39].

Cytoplasmic FGFR-3 expression was observed in upper and middle layers of lining epithelium, stromal fibroblast cells, tumor cells and sometimes in the endothelium of tumor stroma. We found FGFR-3 positivity in 75% cases (81/108) of OSCC, 40.28% cases (29/72) of oral PMOLs and 36.54% cases (19/52) of controls. Our findings of FGFR-3 expression in full thickness of normal epithelium and its significant expression (p<0.008) in carcinomas are supported by the results of Raimondi et al. (2006) [36] in experimental model of hamster cheek pouch. The authors found FGFR-3 expression in normal mucosa as well as its significant expression in carcinoma, predominantly in the basal layers. The presence of FGFR-2 and FGFR-3 receptors in epithelial cells, fibroblasts and endothelia are an evidence to their participation in normal epithelial growth and in the maintenance of connective tissue structures and of vascular network [36].

FGFR-3 has been shown to play an important role in bladder cancer growth and is suggested as a candidate for targeted therapy [40]. In human bladder cancer, the FGFR-3 mutations were observed to be strongly associated with non invasive, low grade and stage. A two pathway model of carcinogenesis (favorable and unfavorable) has been proposed in urinary bladder cancer [41,42]. The favorable pathway is characterized by mutations in FGFR-3 and a clinically unfavorable pathway characterized by mutations in p53. Further the detection of FGFR-3 mutations in urine from patients with FGFR-3 mutations in primary tumor indicating tumor recurrence has also been reported. Thus identification of FGFR-3 mutations may be a potential biomarker of prognosis and recurrence [43,44]. This needs to be studied in oral cancer.

In the present study, we found that the FGFR-3 expression was significantly associated with tumor stage. FGFR-3 expression was observed in 51.85% of cases in stages I and II. Unlike our results, other workers have observed more pronounced staining in stages III and IV [27].

Over expression of a gene can be caused by its amplification or aberrant transcriptional regulation. Increased mRNA levels of *FGF-2* and its receptors were observed in oral cancer and precancer patients as compared to normal controls in the present study. Relative gene expression of *FGF-2* was 28.62 fold higher in OSCC, 21.75 fold in Leukoplakia and 6.79 fold higher in OSMF as compared to healthy controls. *FGFR-2* relative gene expression was 20.21 fold higher in OSCC, 16.79 fold higher in LKP and 9.72 fold higher in OSMF as compared to healthy controls. Relative gene expression of *FGFR-3* was 7.05 fold higher in OSCC, 4.45 fold higher in LKP and 3.95 fold higher in OSMF as compared to healthy controls. Elevated levels of FGFRs have been found in numerous cancers such as cancer of brain, head and neck, lung, stomach, breast, prostate and in sarcomas and multiple myeloma. However, an elevated level of a protein in cancer cell does not necessarily mean that this protein plays a role in carcinogenesis. Further it is not always clear that the FGFR alterations found in human cancers are “drivers” or “passengers” [39].

Several alterations, most often leading to increased FGFR signaling have been associated with human carcinogenesis and development of a malignant phenotype [39]. FGFs/FGFRs are key regulators of mesenchymal-epithelial communication. In adults, FGFR signaling continues to regulate tissue homeostasis and is also involved in processes such as tissue repair, angiogenesis and inflammation. In angiogenesis and neovascularization, FGFR signaling is mainly thought to play an indirect role by influencing other growth factors such as VEGF and hepatocyte growth factor [45].

FGFRs have also been observed in several cancers suggesting a tumor suppressor role of FGFR signaling in these cases. However, it is currently not well understood how FGFR-2 signaling seems to exhibit tumor suppressing effects in some cells and oncogenic effects in others. The basic mechanisms of FGFR signaling have been discussed in an excellent review on roles of fibroblast growth factor receptors in carcinogenesis by Haugsten et al. (2010) [39].

Imbalanced FGFR signaling could contribute to carcinogenesis and could thus be a potent therapeutic target in several human cancers. Several promising FGFR tyrosine kinase inhibitors and FGFR-blocking antibodies have been developed and some of them are in early phases of clinical trials [46, 47].

We found malignant conversion of PMOLs (OSMF, LKP) to OSCC in 13.89% (10/72) cases with mean follow up of 29.41 months. All the precancer cases 7 LKP (16.28%) and 3 OSMF (10.34%) which transformed into malignancy, had associated epithelial severe dysplasia. This number is an under-representation of the population because our study was restricted to only biopsy proven cases of pre-cancerous lesions, that too only in two categories i.e. LKP and OSMF. Further, the cases of OSMF are also limited, as all clinical cases which report to us, have not been included in the study. Only those cases of OSMF who underwent surgery as a part of their treatment were included in our study. FGF-2 was positive in 90% (9/10) and FGFR-2 was positive in 80% (8/10) of transformed cases, a finding of statistical significance (FGF-2 p<.0001; FGFR-2 p<.001). Myoken et al. (1994) [48] in their immunohistochemical study, reported stronger expression of FGF-2 in OSCCs as compared to normal tissue, suggesting the possibility of its involvement in malignant transformation and self proliferation of cells. Fibroblasts transfected with *FGF-2* cDNA underwent malignant transformation thereby acquiring self proliferative ability.

OSMF is an established precancerous lesion prevalent in India [49]. It is caused mainly due to the habit of chewing betel quid which subjects the oral mucosa to direct and frequent contact with chemical carcinogens. One third of OSMF cases slowly progress into squamous cell carcinoma. In experimental carcinogenesis model of oral cancer, when chemical cancerization solution is spread over the mucosa of hamster cheek pouch (one of the most widely accepted animal model of oral cancer), the first change observed is marked desmoplasia or fibrosis underlying the cancerized epithelium, even before the abnormal morphologic epithelial lesions occur. Fibrosis or desmoplasia in the oral cancer may be either a stromal component of the tumor itself or it may be associated with preceding OSMF [36].

The principal cells in OSMF implicated as a source of extracellular matrix in the areas of fibrosis are fibroblasts. Accumulation of connective tissue matrix is secondary to factors such as cytokines and growth factors. In the present study, 3 out of 29 (10.34%) cases of OSMF progressed into OSCC. In a report from southern India, 40% of oral cancer patients had OSMF. Incidence of 7.6% oral cancer in OSMF patients was reported by authors in a median follow-up period of 10-year. We however, had a follow up period of 5 years in this study. Increased FGF-2 expression can be explained due to an initial injury phase because of areca consumption, followed by cellular activation by chemotactic cytokines and other growth factors with eventual fibrosis occurring as a result of molecular alteration at the cellular level [50].

The univariate and multivariate analysis with logistic regression showed statistically significant expression of only FGF-2 and FGFR-2 and their combined co-expression in cases which transformed from precancer into malignancy in the present study. Tumor derived FGF-2 may promote cancer progression by elevating proteolytic enzymes and by paracrine stimulation of vascular endothelial cell growth [51].

Shi et al. (2010) [52] have reported immunohistochemical expression of 2 other modulators of angiogenesis; podoplanin and ABCG2 as markers for evaluating risk of malignant transformation of oral lichen planus. We however, did not have any case of oral lichen planus in our study.

In the present study, presence of dysplasia was found to be a significant clinico-pathologic predictor for malignant transformation in precancer [53]. Age, sex, tobacco, smoking, and alcohol intake were not predictors of malignant transformation. Our findings are in close conformity with previous reports [52, 54].

Our data showed up-regulated FGF-2, FGFR-2 and FGFR-3 expression both at phenotypic and molecular level from PMOLs to OSCC with statistically significant co-expression of FGF2 and FGFR2. Therefore, expression of FGF-2 and FGFR-2 may serve as an adjunct to histopathologic assessment of epithelial dysplasia for evaluating progression and malignant transformation in PMOLs. However, the study is limited by the overall numbers of converted patients. Further studies need to be done in a large cohort to draw a strong conclusion.
